# A novel open-porous magnesium scaffold with controllable microstructures and properties for bone regeneration

**DOI:** 10.1038/srep24134

**Published:** 2016-04-13

**Authors:** Meng-qi Cheng, Tuerhongjiang Wahafu, Guo-feng Jiang, Wei Liu, Yu-qin Qiao, Xiao-chun Peng, Tao Cheng, Xian-long Zhang, Guo He, Xuan-yong Liu

**Affiliations:** 1 Department of Orthopedics, Shanghai Sixth People’s Hospital, Shanghai Jiao Tong University, Shanghai 200233, China; 2 State Key Lab of Metal Matrix Composites, School of Materials Science and Engineering, Shanghai Jiao Tong University, Shanghai 200240, China; 3 State Key Laboratory of High Performance Ceramics and Superfine Microstructure, Shanghai Institute of Ceramics, Chinese Academy of Sciences, Shanghai 200050, China

## Abstract

The traditional production methods of porous magnesium scaffolds are difficult to accurately control the pore morphologies and simultaneously obtain appropriate mechanical properties. In this work, two open-porous magnesium scaffolds with different pore size but in the nearly same porosity are successfully fabricated with high-purity Mg ingots through the titanium wire space holder (TWSH) method. The porosity and pore size can be easily, precisely and individually controlled, as well as the mechanical properties also can be regulated to be within the range of human cancellous bone by changing the orientation of pores without sacrifice the requisite porous structures. *In vitro* cell tests indicate that the scaffolds have good cytocompatibility and osteoblastic differentiation properties. *In vivo* findings demonstrate that both scaffolds exhibit acceptable inflammatory responses and can be almost fully degraded and replaced by newly formed bone. More importantly, under the same porosity, the scaffolds with larger pore size can promote early vascularization and up-regulate collagen type 1 and OPN expression, leading to higher bone mass and more mature bone formation. In conclusion, a new method is introduced to develop an open-porous magnesium scaffold with controllable microstructures and mechanical properties, which has great potential clinical application for bone reconstruction in the future.

Each year over 400,000 bone-grafting operations are performed in Europe and more than 600,000 in the United States, bringing considerable socioeconomic burdens for their healthcare systems^1^. Autograft is deemed as the first choice in bone defect regeneration because it provides osteoconductive scaffolding, osteogenic cells as well as endogenous growth factors and exhibits a high incorporation rate with early revascularization^2^,^3^,^4^. Nevertheless, the use of bone autograft is severely hampered by elevated fracture rates, donor site morbidity and shortage of supply^5^. Therefore, to meet the increased demand for orthopedic implantation, the development of biodegradable porous scaffolds with satisfactory mechanical properties, biocompatibility and osteoinductivity for musculoskeletal tissue engineering is critical^6^. Open-porous magnesium scaffold seems has the potential to serve as an ideal scaffold for bone substitute application due to its relatively excellent mechanical properties and biodegradability^7^,^8^. *In vitro* studies suggest that Mg ions, the biodegradation products of porous magnesium, can promote osteoblast proliferation, differentiation and expression of osteogenic markers^9^,^10^. More importantly, numerous *in vivo* studies have also demonstrated that the porous magnesium scaffolds in endosseous sites can safely degrade and remarkably induce new bone formation and stimulate angiogenesis^7^,^11^,^12^.

Porosity and pore size are crucial morphological properties of a porous scaffold for bone regeneration. Porous scaffold should exhibit porosity in excess of 50% with a minimum recommended pore size of 100 μm^13^,^14^,^15^. What is more, subsequent studies reported that the average pore sizes larger than 300 μm provide better conditions for bone formation and vascularization^16^,^17^. However, on the one hand, highly porous magnesium scaffolds generally exhibit poorer mechanical properties which were significantly influenced by their porous structures (pore size, porosity and the orientation of pores)^1^,^18^,^19^,^20^. To date, numerous methods including powder metallurgy with space holder^18^, directional solidification^21^, and vacuum foaming^22^ have been reported to prepare porous magnesium scaffolds. Unfortunately, those traditional production routes are difficult to accurately control the pore morphologies and obtain appropriate mechanical properties simultaneously. Mechanical strength plays a key role in surgical handling and bearing osteogenic loads during healing. Therefore, the question of how to well balance the adequate mechanical properties and the obligatory porous structure of porous magnesium scaffolds need to be addressed. On the other hand, to the best of our knowledge, no study has so far reported the effects of pore size of porous magnesium scaffold on *in vivo* osteogenesis.

Despite the advantages of porous magnesium scaffolds, the negative issues related to the fast degradation should be solved^8^,^12^. Previous studies have shown that the presence of impurities undermines the corrosion resistance of Mg-based materials as they represent active cathodic sites causing accelerated local micro-galvanic corrosion^23^,^24^,^25^,^26^. Therefore, the purification of porous magnesium scaffolds may be an effective way to improve their corrosion resistance. Additionally, fluoride coating has been applied to the surfaces of different Mg-based alloys by several authors, and the results demonstrated that it could reduce the initial corrosion rates both *in vitro* and *in vivo*^27^,^28^,^29^. Furthermore, other group revealed that Fluoride has beneficial effects on the growth and mineralization of osteoblast *in vitro* and stimulates bone formation *in vivo*^30^,^31^,^32^. Therefore, a homogeneous MgF_2_ coating formed on the surface of high purity porous magnesium scaffolds seems to be able to suppress the rapid corrosion.

In this study, two open-porous magnesium scaffolds with different pore size (250 and 400 μm) were successfully fabricated with high-purity Mg ingots through the titanium wire space holder (TWSH) method. The porosity and pore size could be precisely and individually controlled, as well as the mechanical properties also could be regulated by changing the orientation of pores without sacrificing the requisite porous structure. Meanwhile, MgF_2_ layer was formed on the surface of porous magnesium scaffold during the fabricate process, which can further effectively promote its corrosion resistance. The objectives in present study were to determine the mechanical properties of the novel porous magnesium scaffolds, evaluate the *in vitro* and *in vivo* performances including corrosion behavior, biocompatibility, as well as bone regeneration capacity. Furthermore, the influence of pore size on the *in vivo* bone formation was also investigated.

## Results

### Preparation of open-porous magnesium scaffolds

**Figure 1:**
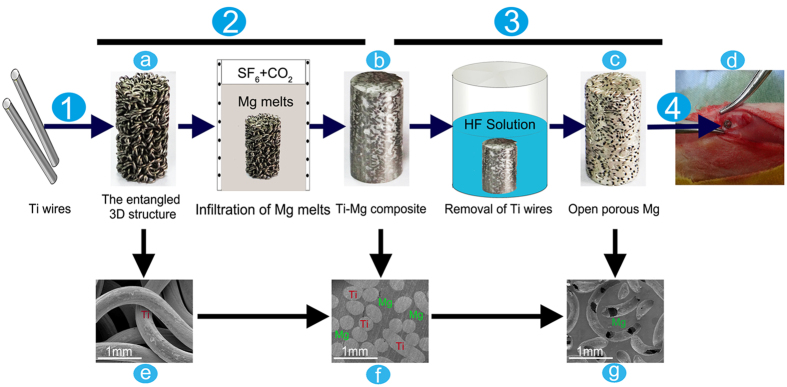
Illustration of preparation process of the open-porous magnesium scaffold and *in vivo* animal model. Step1: the 3D entangled titanium wire material (**a,e**) was prepared with Ti wires. Step2: the Ti-Mg composite (**b,f**) was prepared with high purity Mg melts. Step3: Ti wires were removed by HF solution and open-porous magnesium scaffold(**c,g**) was successful manufactured.Step4:open-porous magnesium scaffolds were implanted into the lateral epicondyle of rabbits(**d**).

Open-porous magnesium scaffolds with varying pore size were successfully synthesized. The schematic representation of the prepared process is shown in Fig. 1. The macroscopical and cross-sectional SEM images of the 3D entangled titanium wire material (Fig. 1a,e) demonstrated a reticulate structure comprising well-distributed Ti wires. The porosity, the shape and the spatial structure of 3D entangled titanium wire material, which are closely correlated with the morphology of porous magnesium scaffolds, could be easily adjusted by wire-woven method. Figure 1b,f shows the morphology of titanium/magnesium composite. Both Ti phase and Mg phase were clearly distinguished, and all the pores in the 3D entangled titanium wire material were occupied by magnesium matrix. As shown in Fig. 1c,g, the morphology of open-porous magnesium scaffold demonstrated that the 3D entangled titanium wire material, as a space holder for the fabrication of porous magnesium, was completely removed by the strong chemical reaction with HF solution.

### Microstructure and surface chemical composition of open-porous magnesium scaffold

**Figure 2:**
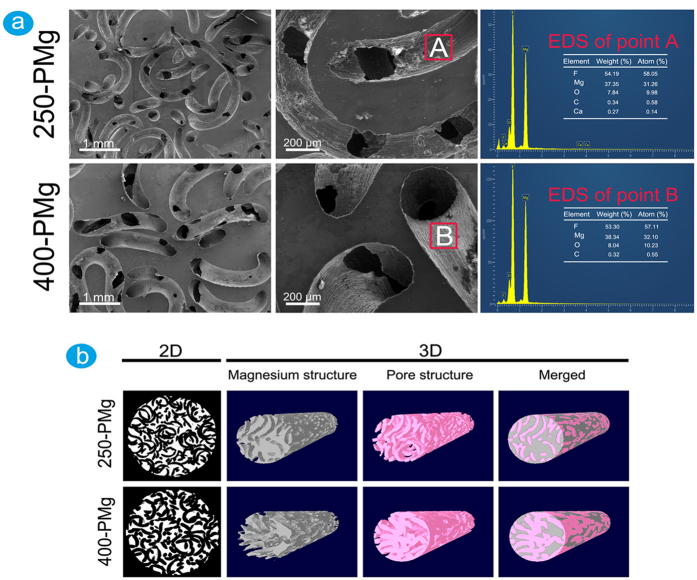
The microstructures and chemical compositions of open-porous magnesium scaffolds. (**a**) SEM and EDS results of 250-PMg and 400-PMg scaffolds; (**b**) 2D and 3D images of 250-PMg and 400-PMg scaffolds acquired from micro-CT scanning.

The morphologies of open-porous magnesium scaffolds respectively prepared by 250 μm and 400 μm Ti wires are shown in Fig. 2a. The pipe-like pores in both 250-PMg and 400-PMg scaffolds were uniformly distributed and interconnected, and the pore size is identical and equals to the diameters of initial Ti wires, indicating that the pore size of porous magnesium can be precisely and readily controlled by selecting different diameter Ti wires. To determine the surface elemental composition, EDS analysis was performed, and the results are shown in Fig. 2a. The surface atomic ratio of F to Mg is approximately 2:1, which coincides well with the atomic ratio in MgF_2_. Therefore it can be reasonably deduced that a protective MgF_2_ coating was formed on both scaffold surfaces.

Micro-CT scanning was conducted to quantify the pore size and the porosity of the scaffold. Figure 2b depicts the 2D cross-sectional and 3D reconstruction images of scaffolds. Both 250-PMg and 400-PMg scaffolds presented roughly homogenous porous structures, and almost all pores were interconnected and opened (Table 1). The measured porosity of 250-PMg scaffold was 54.78 ± 2.67% and 400-PMg scaffold was 54.31 ± 3.10%, respectively, for a target porosity of 55%. Thus, no significant differences were found between the measured and designed target porosity, indicating that the porosity of magnesium scaffolds could be well controlled. The measured pore sizes of 250-PMg scaffold (243 ± 3.41 μm) and 400-PMg scaffold (387 ± 5.16 μm) were consistent with the initial diameters of Ti wires, further confirming that scaffolds could be easily and accurately prepared at a target pore size with reasonable reliability.

### Mechanical properties

The compressive strengths and Young’s moduli of 250-PMg and 400-PMg scaffold were tested. The compressive strengths were 41.2 ± 2.14 and 46.3 ± 3.65 MPa, and Young’s moduli were 2.18 ± 0.06 and 2.37 ± 0.09 GPa, respectively. Apparently, both open-porous magnesium scaffolds with the porosities of 55% exhibit even exceeding Young’s moduli to the cancellous bone (0.01–2 GPa). Also, the compression strengths were within the normal range of human cancellous bone (0.2–80 MPa). Table 2 displays a comparison of the mechanical properties of some representative porous magnesium scaffolds, as well as human bones. In general, open-porous magnesium scaffolds showed comparable or even much better mechanical properties than the other porous Mg scaffolds prepared by various methods. Furthermore, compared with the 250-PMg scaffold, the 400-PMg scaffold exhibited better mechanical properties, which may be attributed to the bigger strut size of the 400-PMg scaffold^33^.

### Determination of *in vitro* corrosion rates

**Figure 3:**
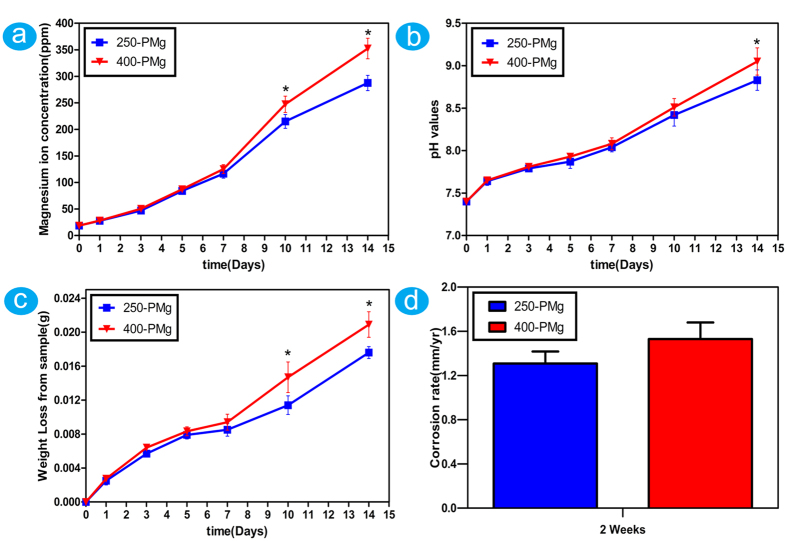
The results of *in vitro* immersion tests of 250-PMg and 400-PMg scaffold. (**a**) Magnesium ions concentration of the immersion extract from 250-PMg and 400-PMg scaffold; (**b**) PH values of the immersion extract from 250-PMg and 400-PMg scaffold; (**c**) Total weight lost from 250-PMg and 400-PMg scaffold; (**d**) The corrosion rates of 250-PMg and 400-PMg scaffolds after immersed for 2 weeks. *Denotes a significant difference between 250-PMg and 400-PMg scaffold (p < 0.05).

Immersion test is a direct way to determine the corrosion resistance. When soaked in the solution, both 250-PMg and 400-PMg scaffolds dissolved gradually with prolonged soaking time. The amount of magnesium ions (Fig. 3a) seems have no big difference between 250-PMg and 400-PMg scaffold in the initial 7 days, which correspond to the variations in pH values (Fig. 3b) and weight loss (Fig. 3c). However, with prolonged immersion time, the 400-PMg scaffold seems degrade faster than the 250-PMg scaffold. Furthermore, in order to quantitatively analyze the differences in degradation between the 250-PMg and 400-PMg scaffold, the *in vitro* corrosion rates were calculated by the weight loss. The 250-PMg and 400-PMg scaffolds showed corrosion rates of 1.31 ± 0.11 mm/yr and 1.53 ± 0.15 mm/yr, respectively, without any big difference between them.

### Cell morphology and attachment

**Figure 4:**
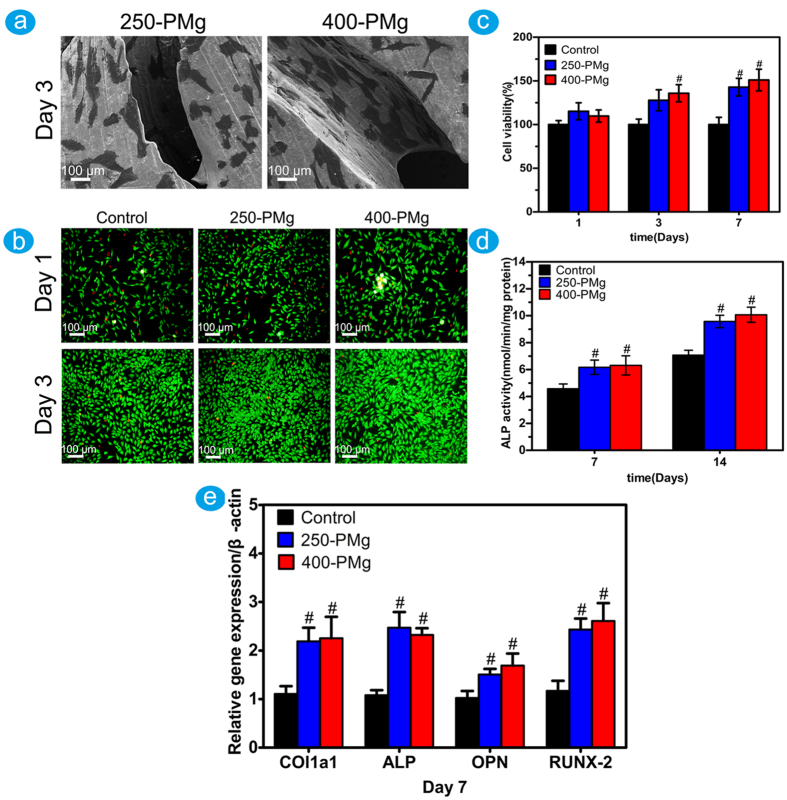
(**a**) Cell morphology on 250-PMg and 400-PMg scaffold after 3 days of incubation displayed by SEM; (**b**) Viability of MG63 osteoblasts in 250-PMg and 400-PMg scaffolds extraction using live/dead assay; (**c**) Viability of MG63 osteoblasts in 250-PMg and 400-PMg scaffold extraction using MTT assay; (**d**) ALP activity of MG63 osteoblasts after 7 and 14 days; (**e**) Osteogenic differentiation of MG63 osteoblasts by measuring the mRNA expression level of Col1a1,ALP, OPN and RUNX-2 after 7 days. ^#^Denotes a significant difference compared to the control (p < 0.05).

The typical SEM images of the cells on the scaffolds are shown in Fig. 4a. After 3 days of cultivation, cells presented a flattened morphology and grew well on both surfaces of 250-PMg and 400-PMg scaffolds.

### Indirect cytotoxicity and cell differentiation

The indirect cytotoxicity of the scaffolds was evaluated by live/dead staining using the extracts of the scaffolds. As shown in Fig. 4b, nearly all cells were viable during cultivation in each group, and cell densities in the extracts of 250-PMg and 400-PMg scaffold were higher than that in the control group. The upper results were further confirmed by MTT assay. As shown in Fig. 4c, the cell viability of MG63 osteoblasts after culturing in the 250-PMg and 400-PMg scaffold extracts for 7 days, was significantly higher (p < 0.05) than that in the medium without extracts, which meant great stimulation of both 250-PMg and 400-PMg scaffold extracts to the proliferation of MG63 cells. Our results suggested that both 250-PMg and 400-PMg scaffolds coated with MgF_2_ layer possess acceptable cytocompatibility.

To investigate the effect of open-porous magnesium scaffolds on osteoblasts differentiation, the ALP activity and differentiation-related genes were measured using the scaffold extracts. As shown in Fig. 4d,e, compared with the control group, both 250-PMg and 400-PMg scaffolds showed higher ALP activities and increased mRNA expressions of ALP, Runx2, OPN and Col1a1,indicating enhanced differentiation in the extracts of the scaffolds.

### Subcutaneous and intramuscular inflammatory response of open-porous magnesium scaffolds

**Figure 5:**
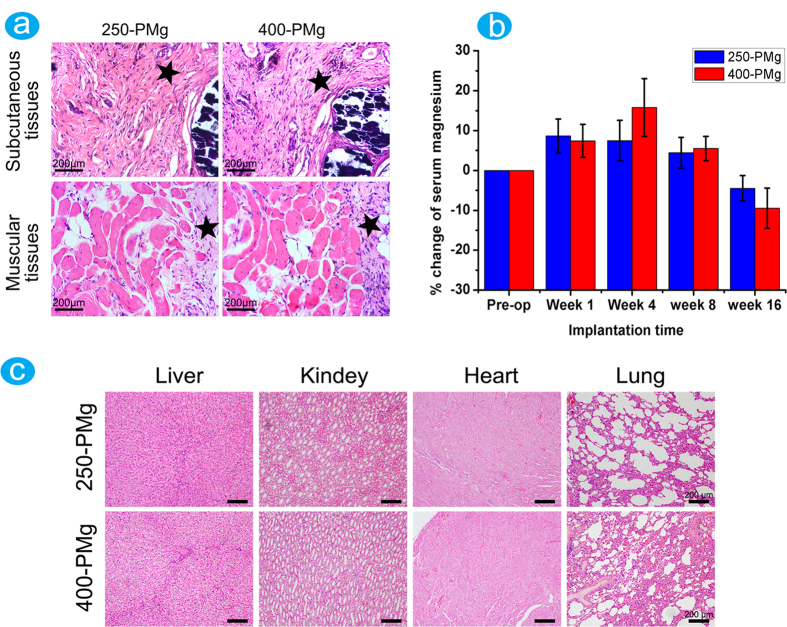
(**a**) H&E staining of subcutaneous and muscular tissue in contact with 250-PMg and 400-PMg scaffold, the pentagrams represent the fibrous tissues formed around the scaffolds; (**b**) Percentage changes in serum magnesium levels before and after implantation; (**c**) Representative sections from important internal organs of the rabbits after implantation with 250-PMg and 400-PMg scaffold for 16 weeks.

Figure 5a shows the histological images of the localized sites of implants harvested from subcutaneous and intramuscular tissues surrounding 250-PMg and 400-PMg scaffolds after 8 weeks. Although fibrous tissues containing a mild population of agglomerated cells and macrophages were observed surrounding the two scaffolds, the parameters for inflammation examined for all implants in this study did not exceed the moderate level.

### Important internal organs histology observation and serum magnesium ion concentration measurement

Figure 5b shows the percentage changes in the serum magnesium levels of rabbits during the 16 weeks post implantation. The serum magnesium concentrations in the rabbits implanted with either 250-PMg or 400-PMg scaffold fluctuated between −9.5% and 15.8%, which is not significant.

Histological sections of important organs, including the heart, liver, lungs, and kidneys are shown in Fig. 5c. No obvious pathological changes were found in these organs at 16 weeks after implantation.

### Micro-computed tomography analysis

**Figure 6:**
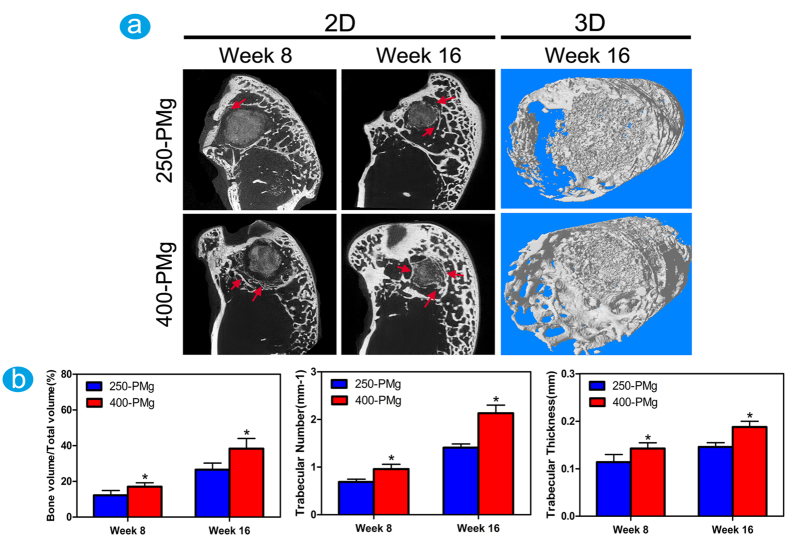
Characterization of scaffolds and the newly formed bone by Micro-CT. (**a**) Micro-CT 2D (The red arrows refer to the newly formed bone) and 3D reconstruction models showing the status of new bone (white in color) response 16 weeks after surgery; (**b**) Quantitative analysis of bone volume fraction (BV/TV),trabecular number (TN) and trabecular thickness. *Denotes a significant difference compared to the 250-PMg scaffold (p < 0.05).

Micro-CT was used to observe possible changes in the residual scaffold’s volume and density, as well as bone formation adjacent to the degrading magnesium scaffolds. The two-dimensional and three-dimensional images (Fig. 6a) reveal that the volumes and densities of both the scaffolds decreased after 8 weeks of implantation. After 16 weeks of implantation, almost all the implanted scaffolds were vanished and replaced by newly formed bone. In addition, it must be point out that the 250-PMg scaffold seems degrade slowly than the 400-PMg scaffold. As for the new bone formation, on the 400-PMg side, significantly more regenerated bone was presented than on the 250-PMg side at both time points, meanwhile, the bone directly neighboring or in the implant sites shows a higher density. Consistently, the quantitative assessments of bone volume fraction (BV/TV), trabecular number (TN) and trabecular thickness (TH) were determined in a sequence of 400-PMg >250-PMg at both time points (Fig. 6b), indicating a more densely packed bone structure around the 400-PMg scaffold. Furthermore, No gas cavities were found at both time points in the two groups.

### Histological and immunohistological analysis

**Figure 7:**
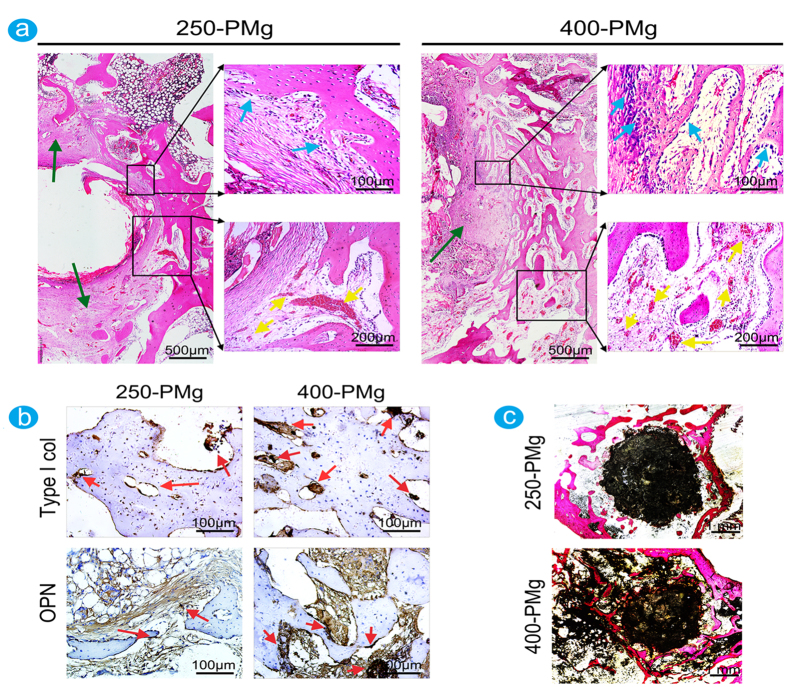
Histological and immunohistological analysis of bone formation at 8 weeks after the implantation. (**a**) Representative histological analysis (H&E staining) of bone formation in the 250-PMg and 400-PMg scaffold group. The lower images are higher magnifications of the areas within the black boxes. In the 250-PMg scaffold group, a fibrotic walling-off phenomenon based on the formation of regular dense fibrous tissues (green arrows) with rare inflammatory cells was observed, whereas less fibrous tissue accompanied with more lymphocytes, macrophages and foreign body giant cells (FBGCs)were present around the 400-PMg scaffold. Compared with the 250-PMg scaffold, more new bone formed around 400-PMg scaffold, and more abundant cuboid osteoblasts (blue arrows) were observed between the fibrous tissue and new bone. Furthermore, in the 400-PMg scaffold group, more newly blood vessels (yellow arrows) formed than in the 250-PMg scaffold group. (**b**) Relatively higher expressions of collagen type 1 and OPN (red arrows) in the 400-PMg scaffold group than that in the 250-PMg scaffold group. (**c**) Representative histological analysis (Van Gieson’s picrofuchsin staining) of bone formation in the 250-PMg and 400-PMg scaffold group. Significantly more new bone formed in the 400-PMg scaffold group was observed.

Histological analysis was performed to give more detailed analysis on the bone response to the scaffolds with different pore size. As shown in Fig. 7a, at 8 weeks after the implantation, 250-PMg scaffold induced a fibrotic walling-off phenomenon based on the formation of regular dense fibrous tissues with rare inflammatory cells. In contrast, relatively less fibrous tissue accompanied with more lymphocytes, macrophages and foreign body giant cells (FBGCs) were present around the 400-PMg scaffold. These findings indicated that the 400-PMg scaffold induced a relatively severe inflammatory reaction. Interestingly, however, compared with the 250-PMg scaffold, there were more new bone formed around the 400-PMg scaffold and more abundant prominent cuboid osteoblasts lay on the osteoid seams. Moreover, more new born blood vessels were observed in the 400-PMg scaffold group. The expressions of the collagen type 1 and OPN was visualized by immuohistochemical staining (Fig. 7b). In the 250-PMg scaffold group, moderate collagen type I and OPN expression were observed, while in the 400-PMg scaffold group, strong collagen type I and OPN signals were observed at the boundary of the newly formed woven bone. Figure 7c shows the non-decalcified tissue staining. The scaffolds were adsorbed and the new bone invaded the scaffolds from the edge to the center. Moreover, 400-PMg scaffold promoted more new bone formation, and the residual material volumes showed as just the opposite situation: the residual 400-PMg scaffold was clearly less than that of the 250-PMg scaffold, indicating a relative faster degradation of the 400-PMg scaffold.

**Figure 8:**
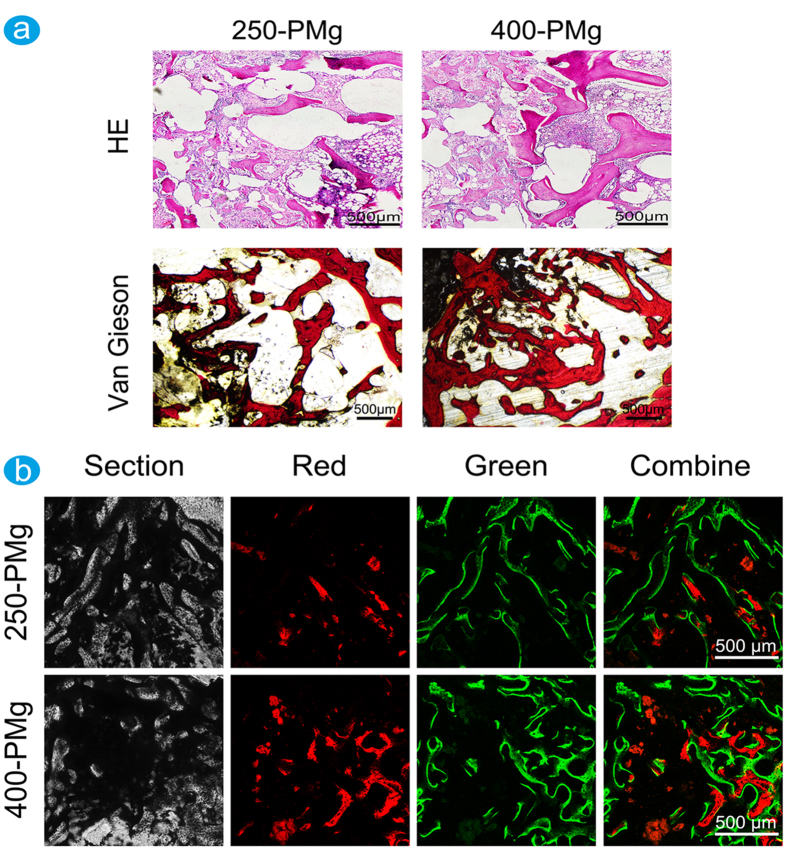
Histological and sequential polychrome labels analysis of bone formation at 16 weeks after the implantation. (**a**) Representative histological analysis (H&E staining and Van Gieson’s picrofuchsin staining) of bone formation in the 250-PMg and 400-PMg scaffold group. The inflammatory responses to both 250-PMg and 400-PMg scaffolds recede with progressive time after implantation and there are more bone formed in the 400-PMg scaffold group; (**b**) Sequential polychrome labels observed for 16 weeks in rabbit models: alizarin red S (red) and calcein (green).

At 16 weeks after the implantation, as shown in Fig. 8a, the two scaffolds were almost degraded completely and replaced by newly formed bone and the inflammatory responses to both 250-PMg and 400-PMg scaffolds were alleviated with progressive time after implantation. What is more, compared with 250-PMg scaffold, more new bone tissues are formed in the implant site of 400-PMg scaffold. The upper results were further confirmed by the sequential fluorescent labeling. As shown in Fig. 8b, stronger density and more widely spread area of Alizarin Red S (red) and calcein (green) fluorescence were observed in the 400-PMg scaffold group than that in the 250-PMg scaffold group, which demonstrated that open-porous Mg scaffold with larger pore size stimulated more new bone formation.

## Discussion

An ideal porous scaffold for bone tissue engineering should have certain properties including high levels of porosity, appropriate pore size, and interconnected pore network to offer adequate spaces for the ingrowth of cells and surrounding tissues and nutrient transport^34^. Most importantly, in addition to the necessary porous structure, adequate mechanical properties similar to that of cancellous bone should be guaranteed in terms of reducing the premature failure of the implants^9^. In this study, we report a new approach to fabricate a well-controlled 3D open-porous magnesium scaffold. The results suggest that the pore size and the bulk porosity of the porous magnesium scaffold could be easily, precisely and individually controlled. Furthermore, the mechanical properties of porous magnesium scaffold partly determined by the spatial structure could be tailored by regulating the orientation and distribution of the Ti wires, which makes it feasible to acquire the adequate mechanical properties and the obligatory porous structure at the same time. Therefore, this method for the production of open-porous Mg implants has significant advantages compared with other methods reported previously.

The corrosion behaviors of the scaffolds were also investigated in this work. Based on the weight loss after an immersion time of two weeks, the corrosion rates of the 250-PMg and 400-PMg scaffolds were estimated at 1.31 ± 0.11 mm/yr and 1.53 ± 0.15 mm/yr, respectively. Such corrosion rates, however, were even lower than that of the pure magnesium disk with a purity of 99.9% reported by Yunchang Xin *et al.*^35^. One possible reason for the difference may be due to the purity of magnesium (99.98%) used in this study, which might affect the corrosion rate by 2 orders of magnitude^36^. Song *et al.* have also demonstrated that the corrosion rate is low when the impurity concentration is lower than the tolerance limit^24^. On the other hand, the changing trends of magnesium ions, pH values and weights loss in the immersion test suggest that both 250-PMg and 400-PMg scaffolds degrade slowly and have no big difference in the first one week, which may be attributed to the initial protective action of the MgF_2_ coatings. These observations are consistent with a previous *in vivo* study which demonstrated that MgF_2_ coatings possess the ability to reduce the initial corrosion rate of LAE442 and, temporarily, the release of alloying elements^27^. Moreover, clearly visible gas accumulation is usually considered as a sign of too fast implant degradation and several studies have also documented gas cavity formation near Mg based implants *in vivo*^7^,^27^,^37^. Whereas no gas cavities were observed in this study based on our micro-CT results, indicating that the MgF_2_ coating on the high purity porous Mg reduced the initial corrosion rate and limited the local hydrogen release.

The *in vitro* biocompatibility was determined by direct cell attachment and indirect cytotoxicity tests. Cells grew very well on the scaffolds and no obvious toxic effects were observed, which is consistent with the enhanced corrosion resistance of Mg alloys resulting in better cell attachment and growth, as well as higher indirect cell viability as reported in a previous study^38^. Scaffolds possess osteogenic differentiation properties is critical for bone regeneration. In this study, the enhanced ALP activity and gene expression of Runx-2, ALP, Col1a1 and OPN in both 250-PMg and 400-PMg extracts suggest that they are able to promote the cell differentiation. These results are further validated by Pei Han *et al.*^23^, who reported that a higher level of ALP activity and up-regulation of bone-related genes in the HP Mg screw extraction.

Local soft tissue response to the presence of the foreign material, particularly the nature and magnitude of the inflammation, is commonly used to evaluate the *in vivo* biocompatibility. In the present study, although vascularization, fibrous tissue, and certain amounts of foreign body giant cells were noted in the histological sections studied, the inflammation parameters in both 250-PMg and 400-PMg groups did not exceed the moderate level and severe inflammatory reactions were not observed in any of the tissues. This suggests that the implants are tolerated *in vivo* and they have the potential for use as a biodegradable implant material. Furthermore, even though the excess amount of magnesium ions, not absorbed by the body, can be excreted in the urine and side effects caused by excess amounts of magnesium ions in body are rare^9^. It was still necessary to monitor the potential adverse effects of Mg ions and evaluate their biological safety. Our results confirmed that there were no obvious pathological changes in the internal organs after 16 weeks implantation and all the serum magnesium levels are within the normal physiological ranges throughout the implantation period, indicating that both 250-PMg and 400-PMg scaffolds can safely degrade *in vivo* and will not produce deleterious effects.

In earlier *in vivo* studies, porous magnesium scaffolds have been demonstrated that can enhance bone formation. However, studies investigating bone response to open-porous magnesium scaffolds with different pore size are scarce. In this study, based on the analysis of micro-CT and histology, we found that under similar porosity, magnesium scaffolds with larger pore size elicit a higher level of osteoblastic activity and up-regulated collagen type 1 and OPN expression, leading to higher bone mass and more mature bone formation in the implant site. Although the specific mechanisms underlying these differences are not fully understood, we can get some potential information to illustrate that according to our results.

A very interesting aspect of the effect of pore size on bone regeneration is the impact on vascularization. While recent studies indicated that promoting angiogenesis may be not the only determinant for successful bone remodeling^4^,^39^, metabolically active osteogenic cells within the implanted sites definitely require a sufficient supply of oxygen tension, essential trace elements and nutrients for direct osteoneogenesis^40^. Additionally, stimulation of vessel formation using angiogenic factors can augment bone formation and fracture healing in model systems have been demonstrated^41^,^42^. Thus, the higher vascularization surrounding the 400-PMg scaffold in this study indicates that larger pore size can induce more new blood vessels, which may be partially responsible for the enhanced bone formation. This result is further confirmed by Kuboki Y *et al.*^43^, who showed that the enhanced vascularization and direct osteogenesis were observed with hydroxyapatite scaffolds containing larger tunnel (350 μm) diameters.

In addition to the porous structure, the Mg ions, which are the by-product of biodegradable scaffolds, are critical to the regulation of bone regeneration. In this study, due to the presence of Mg ions, collagen type 1 and OPN, the prerequisite for extracellular matrix formation and mineralization in bone, were up-regulated at the gene level and this effect has been demonstrated by various *in vitro* studies^9^,^10^. Furthermore, as magnesium is a key component of the ribosomal machinery that translates the genetic information encoded by mRNA into polypeptide structures^44^, thus, it could be inferred that the protein translation for the extracellular matrix such as collagen type I and OPN might be enhanced by high concentrations of magnesium^45^. And, coincidently, enhanced expression of collagen type 1 and osteopontin around fast corroding Mg implants *in vivo* has been observed^12^,^46^.

Taking the upper two factors into consideration, it is reasonable to believe that as 400-PMg scaffold degrade *in vivo*, owing to the larger pore size can promote the exchange of body fluid and vascularization, magnesium ion is released and extensively diffused to the surrounding tissues, recruiting osteoblasts and mesenchymal stem cells from the bone marrow and the vasculature to the implant surface^47^, stimulating the synthesis and accumulation of ECM, leading to an enhanced osteoid deposition. Moreover, the different foreign body reactions to 400-PMg and 250-PMg scaffold should also be taken into consideration. In the present study, a large amount of lymphocytes, macrophages and foreign body giant cells (FBGCs) and small areas of fibrous tissue were observed around the 400-PMg scaffold at 8 weeks, in contrast to the 250-PMg scaffold showing large areas of fibrous tissue with rare lymphocytes, macrophages and FBGCs. This may be attributed to the porous magnesium scaffold containing larger pores has a relative high corrosion rate, and a strong inflammatory response. Traditional concept always simply regards the presence of FBGCs represent an inferior environment for bone formation^48^, which is controversial with our results that more new bone formed in the implant site of the 400-PMg scaffold. Furthermore, as implanted time elapsed and the scaffold degraded, the regression of FBGCs was observed in this study at 16 weeks. Therefore, we believe that FBGCs are not a sign of pathology and does not impair bone formation. The walling-off phenomenon may be another reason to illustrate the differences in bone response to the two scaffolds. The large areas of fibrous tissue formed surrounding 250-PMg scaffold may indeed downsize the foreign body reactions, but also restrict the angiogenesis and diminish nutrient supply that maybe result in inactive osteogenetic reaction^37^,^49^. However, it must be point out that the foreign body reactions to implanted materials in regulating bone regeneration are complex and the specific mechanism need for further study.

## Conclusion

The open-porous magnesium scaffolds were successfully fabricated using high-purity Mg ingots through the TWSH method. The porosity and pore size can be precisely and individually controlled, as well as the mechanical properties can also be regulated to be within the range of human cancellous bone by changing the orientation of pores without sacrifice the requisite porous structures. Meanwhile, MgF_2_ layer was uniformly formed on the surface of the porous magnesium scaffold during the fabrication process. MgF_2_ layer, function as a barrier coating for corrosion protection, formed on the high purity porous magnesium can help reduce the hydrogen release and toxicity (local or systemic) response caused by the initial high corrosion rate of magnesium. Cells can grow and proliferate well in the extractions of the open-porous magnesium scaffolds, indicating the good cytocompatibility of the scaffolds. The enhanced ALP activity and expression of osteogenic differentiation related genes suggest that the scaffolds can stimulate new bone formation. Moreover, the *in vivo* animal studies show a moderate local inflammation response and no systemic toxicity. Under the same porosity, magnesium scaffolds with larger pore size can promote vascularization and up-regulated collagen type 1 and OPN expression, leading to higher bone mass and more mature bone formation in a rabbit model.

## Materials and Methods

### Preparation of open-porous magnesium scaffolds

Open-porous magnesium scaffolds with two different pore sizes were synthesized using TWSH method^50^. The scaffolds were prepared through three steps, as shown in Fig. 1. First, the 3D entangled titanium wire materials were prepared by using different diameter titanium wires (250 μm and 400 μm), which were used as space holder materials. In this step, the porosities of the two scaffolds were determined at almost 55% through control the porosities of the initial entangled 3D structures. Then the 3D entangled titanium wire materials were soaked in the magnesium melts (purity: 99.98%), keeping at 700 °C under the atmosphere of the mixed gas of SF_6_ and CO_2_, and the supersonic vibration (the source frequency: 40 kHz; output: 80 W; the vibration time is 60 s) was applied to improve the melt infiltration into the porous structures of the 3D entangled titanium wire materials. The titanium/magnesium composites were formed in this step. Eventually, the specimens were immersed in the 40% hydrofluoric acid at room temperature in an ultrasonic bath to remove the 3D entangled titanium wire material. When all the titanium wires in the composites were etched off, the specimens were subsequently immersed in the 40% hydrofluoric acid for 96 h. During this step two open-porous magnesium scaffolds with pore size 250 μm (Denoted as 250-PMg in this work) and 400 μm (Denoted as 400-PMg in this work) were successfully manufactured, while a magnesium fluoride layer was formed on the magnesium surface. More detailed process was described in our previous study^51^. Φ10 mm × 20 mm samples were prepared for mechanical tests and Φ3 mm × 5 mm samples were prepared for *in vitro* and *in vivo* tests. All samples were ultrasonically cleaned with acetone, absolute ethanol and distilled water for 10 min each and then sterilization with ethylene oxide.

### Characterization of open-porous magnesium scaffolds

The scaffold morphology was observed using scanning electron microscopy (FESEM, FEINOVA NanoSEM), and the surface coating chemical composition was characterized via energy dispersive X-ray spectroscopy (EDS, Oxford). For an exact characterization of the basic properties of the scaffolds, each sample was scanned in a micro-computed tomography device (SKYSCAN 1176, Bruker). The two-dimensional (2D) and three-dimensional (3D) models were reconstructed using the NRecon (Skyscan Company) and CTVol (Skyscan Company), and furthermore, the pore size, the porosity, the initial surface area and other basic features were determined.

### Mechanical testing

The compression strength and Young’s modulus of the scaffolds were measured in compression in a Zwick AG-100KN testing machine. The tests were conducted on the specimens with dimensions of Φ10 mm × 20 mm at a cross-head speed of 1 mm/min.

### *In vitro* corrosion resistance test

Immersion test for the two scaffolds were carried out in a solution of media-volume–to-surface-area ratio of 50 ml cm^−2 ^52. Six each of the 250-PMg and 400-PMg samples were individually immersed in DMEM containing 10% fetal bovine serum at 37 °C for 14 days. The concentration of Mg ions was measured at six different time points of 1, 3, 5, 7, 10 and 14 days by inductively coupled plasma-atomic emission spectroscopy (ICP-AES; Perkin Elmer Optima 2000 DV). Furthermore, the pH values were monitored and the weight loss of the samples was measured after removal of the corrosion products in chromic acid at each point. The average corrosion rate (CR) for the Mg scaffolds was calculated as follows:


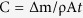


Where C is the corrosion rate in mm year^−1^, ∆m is the weight loss, ρ is the density of the material, A is the initial implant surface area and t is the immersion time.

### Cell culture

With consideration of the potential use of the Mg scaffold in the orthopedic field, the human MG63 osteoblasts were used in this study. MG63 cells were cultured in Dulbecco’s modified Eagle’s medium (DMEM, Gibco) supplemented with 10% fetal bovine serum (Gibco) in a humidified atmosphere of 5% CO_2_ at 37 °C.

### Direct cell attachment on the open-porous magnesium scaffolds

MG-63 cells (1 × 10^4^ cells/well) were seeded on the two scaffolds. After 3 days of incubation, Samples were fixed by 2.5% glutaraldehyde solution and then subjected to step dehydration in ethanol. After coating gold, the samples were observed under a field emission scanning electron microscope (FESEM, FEINOVA NanoSEM).

### Preparation of biomaterial extracts

Scaffolds were immersed into Dulbecco’s modified Eagle’s medium (DMEM, Gibco) containing 10% fetal bovine serum (Gibco) with a surface area to medium volume (1.25 cm^2^/ml) for preparing extracts according to ISO 10993 Part 12 at 37 °C in a humidified atmosphere of 5% CO_2_ for 24 h. The pH values and the concentrations of Mg ions in the extracts were measured by ICP-AES. The extracts were collected without any filtration for further cell tests.

### Indirect cell cytotoxicity

The cytotoxicity of the extracts was evaluated by live/dead staining. Briefly, MG-63 cells (1 × 10^4^ cells/well) were carefully seeded in 24-well plates. After 1 day of incubation, the medium was replaced with the extracted medium and incubated for further 1 and 3 days. Finally, the samples were assessed using a live/dead Kit (Invitrogen) and observed under a fluorescence microscope.

MTT assay was used to further assess the cytotoxicity. MG-63 cells (1 × 10^4^ cells/well) were incubated in 96-well plates. Culture medium without extracts served as the control group. After 1 day incubation, the medium was replaced by the extracted medium and incubated for further 1, 3 and 7 days. 10 μl of MTT solution was added to each well for 4 h and then 100 μl of 10% sodium dodecyl sulfate in 0.01 M hydrochloric acid was added to each well overnight. Finally, the spectrophotometric absorbance of each well was measured using a microplate reader at 570 nm with a reference wavelength of 640 nm.

### Alkaline phosphatase (ALP) activity

The differentiation behavior of MG-63 cells was estimated by measuring ALP activity. MG-63 cells (1 × 10^4^ cells/well) were seeded in 24-well plates and cultured for 1 day. Then the medium was replaced with the extracted medium and incubated for further 7 and 14 days. The cells in each well were washed with phosphate-buffered saline (PBS) and lysed in 0.1% Triton X-100. Then, the lysate was incubated with p-nitrophenyl phosphate (pNPP) (Sigma) for 60 min at 37 °C. Finally, the reaction was quenched by 1 M NaOH solution, the quantity of p-nitrophenol produced was measured at 405 nm and the total protein content was acquired with the aid of a BCA Protein Assay Kit (Sigma).

### Real-time quantitative RT-PCR analysis

Cells cultured in extracts were collected after incubation for 7 days to evaluate the gene expressions of ALP, Runx2, Col1a1 and OPN by the real-time reverse-transcriptase polymerase chain reaction (Real-time RT-PCR). The total RNA was extracted using a TRIzol reagent (Invitrogen). Subsequently, the complementary DNA (cDNA) was synthesized using Reverse Transcriptase M-MLV (Takara). Quantifications of the selected genes were performed using Real-time PCR with SYBR Premix Ex Taq (Takara). The primers used in this study are listed in Table 3, and the β-actin as the internal control gene.

### Surgical procedures

The experimental protocol was approved by the Animal Care and Experiment Committee of Sixth Peoples Hospital affiliated to Shanghai Jiao Tong University, School of Medicine, and all procedures were strictly carried out in accordance with the approved guidelines. Thirty female and adult New Zealand white rabbits with a body weight of 3.87 ± 0.31 kg were randomly divided into two groups corresponding to 250-PMg and 400-PMg scaffold. The rabbits were anesthetized with pentobarbital (25 mg/kg) by intravenous injection. For the surgical procedures, A 3 mm diameter and 8 mm deep hole was made by a hand driller at the lateral epicondyle by a minimally invasive approach (Fig. 1d). Subsequently, the scaffolds (3 mm diameter and 5 mm height) were implanted into the prepared holes, and then the surgical wound was closed carefully. The animals were sacrificed by an overdose of intravenous pentobarbital sodium 8 and 16 weeks following implantation. Additionally, in order to observer the tissue response, the scaffolds were implanted into the lumbar musculature as well as subcutaneously on each side of the back via an axial access 2 cm cranial to the pelvic bone, and the animals were sacrificed after 8 weeks using a high dose of pentobarbital sodium.

### The subcutaneous and intramuscular inflammatory response of open-porous magnesium scaffolds

The scaffolds were removed along with surrounding connective tissue. Samples were fixed in 10% formalin solution. The tissues were cut in 5 μm thick sections, stained with haematoxylin and eosin. Finally, the stained sections were observed using a Leica microscope.

### Assessment of systemic toxicity

Blood was collected prior to surgery, and at 1, 4, 8 and 16 weeks post-operation to determine the magnesium ion concentration. The blood was centrifuged at 3500 rpm for 10 min at room temperature. The serum magnesium ion concentration was determined using ICP-AES (ICP-AES; Perkin Elmer Optima 2000 DV) and the concentrations in the animals with 250-PMg and 400-PMg scaffolds were compared. Furthermore, important internal organs (kidney, liver, lung, heart) were harvested after implanted for 16 weeks and haematoxylin and eosin staining were conducted to observe the possible change.

### Micro-CT assay

At 8 and 16 weeks post-operation, five scaffolds in the explanted bones in each group were scanned in the Micro-CT (SKYSCAN 1176, Bruker). After the 2D planes were reconstructed using the NRecon (Skyscan Company), the 3D models were generated by CTVol (Skyscan Company). To quantify the new bone formed, a region with a radius of 2 mm from the implant was selected for analysis. Bone volume fraction (Bone volume/Total volume, BV/TV), trabecular thickness (Tb. Th) and trabecular number (Tb. N) of new bone were analyzed.

### Histological and immunohistochemical observation

Five femur condyles in each group were fixed in 10% formalin for 2 days, then decalcified using 10% EDTA solution (pH 7.4) for 21 days, finally embedded in paraffin. 5 μm sections of each specimen were collected. Hematoxylin and eosin (H&E) staining was implemented to examine the morphology.

After the deparaffinized sections were prepared, Immunohistochemistry was performed as previously describe^53^. Briefly, the sections were incubated with primary collagen I antibody ((NB600-450, Novus Biologicals, USA) and osteopontin (OPN) antibody (clone 1B20; Novus Biologicals, USA) at 4 °C for overnight, and then incubated with goat anti-mouse biotin-conjugated IgG (Vector Lab Inc, USA) for 1 h. Subsequently, color reaction was developed with diaminobenzidine (Dako, USA). Finally, the sections were counterstained with hematoxylin and examined under a Leica microscope.

The other five femur condyles in each group were fixed in acetone for 7 days, dehydrated in graded series of alcohol for 3 days each, and embedded in methyl methacrylate without decalcification. The non-decalcified sections, which were parallel to the long axis of the femur, were made on a diamond saw (Leica SP1600) and grounded to about 50 μm in thickness. Finally, the specimens were stained with Van Gieson’s picrofuchsin staining.

### Double Fluorescence Labeling

Fluorescent labeling method has been widely used to assess the location and process of new bone formation. In this study, the new bone formed was double labeled by alizarin red (30 mg/kg) and calcein (20 mg/kg) (Sigma), which were intraperitoneally injected at 8 and 12 weeks after the operation, respectively. Finally, the fluorochrome markers were evaluated using a confocal laser scanning microscope (CLSM, Leica) after tissue processing.

### Statistical analysis

The data were presented as means ± standard deviations. Differences between two groups were tested using the Student’s t test. For differences between groups, one-way ANOVA and Student-Newman-Keuls post hoc tests were performed. The differences were considered statistically significant at p values less than 0.05.

### Ethics statement

All animal experiments in this study were approved by the Animal Care and Experiment Committee of Sixth Peoples Hospital affiliated to Shanghai Jiao Tong University, School of Medicine. All procedures were carried out in accordance with the approved guidelines.

## Additional Information

**How to cite this article**: Cheng, M.-q. *et al.* A novel open-porous magnesium scaffold with controllable microstructures and properties for bone regeneration. *Sci. Rep.* **6**, 24134; doi: 10.1038/srep24134 (2016).

## Figures and Tables

**Table 1:** Basic properties of the two scaffolds determined by Micro-CT.

	Unit	250-PMg	400-PMg
Pore size	μm	243 ± 3.41	387 ± 5.16
Total porosity	percent	54.78 ± 2.67	54.31 ± 3.10
Open porosity	percent	54.69 ± 2.14	54.05 ± 2.78
Surface area	mm^2^	235.98 ± 7.47	204.50 ± 8.01
Trabecular thickness (strut size)	mm	0.28 ± 0.04	0.36 ± 0.02
Trabecular number	1/mm	1.65 ± 0.06	1.07 ± 0.01

All data shown as average ± standard deviation.

**Table 2:** Summary of mechanical properties of some representative porous Mg in comparison to those of human bones.

Materials	Porosity (%)	Pore size (μm)	Compressive strength(MPa)	Young’s modulus (GPa)	Reference
Porous Mg	54.78 ± 2.67	250	41.2 ± 2.14	2.18 ± 0.06	Present work
	54.31 ± 3.10	400	46.3 ± 3.65	2.23 ± 0.09	
Cancellous bone	/	/	0.2–80	0.01–2	54
Porous Mg	35–55	70–400	12–17	0.8–1.8	18
Porous Mg	34–54	/	11.1–30.3	0.09–0.39	55
Porous Mg	43–51	/	8–13	0.41–0.63	56
Porous Mg	28	170	24	/	19

**Table 3:** Real-Time Polymerase Chain Reaction Primers Used in This Study.

Gene	Accession number	Primers (F, forward; R, reverse)	Product size (bp)
β-actin	NM_001101.3	F:CCCAAGGCCAACCGCGAGAAGATG	219
		R:GTCCCGGCCAGCCAGGTCCAGA	
OPN	NM_001040058.1	F:TCTGATGAATCTGATGAACTGGTC	195
		R:GGTGATGTCCTCGTCTGTAGCA	
Col 1a1	NM_000088.3	F:ACCTCCGGCTCCTGCTCCTCTTAG	235
		R:GCGCCGGGGCAGTTCTTGGTCT	
ALP	NM_014476.5	F:GACAATCGGAATGAGCCCACAC	222
		R:GTACTTATCCCGCGCCTTCACCAC	
Runx2	NM_001024630.3	F:TGCGGCCGCCCCACGACAA	200
		R:ACCCGCCATGACAGTAACCACAGT	
